# Reducing Chronic Spine Pain in an Adult Male by Decreasing Lumbar Scoliosis and Increasing Cervical Lordosis Using Chiropractic BioPhysics® Protocols: A 26-Month Follow-Up Case Report

**DOI:** 10.7759/cureus.68393

**Published:** 2024-09-01

**Authors:** Jason W Haas, Miles O Fortner, Thomas J Woodham, Deed E Harrison

**Affiliations:** 1 Research, Chiropractic BioPhysics (CBP) NonProfit, Eagle, USA; 2 Chiropractic Biophysics, Western Plains Chiropractic, Gillette, USA

**Keywords:** lumbar lordosis, cbp® rehabilitation, spine rehabilitation, standard radiography, coronal balance, sagittal balance, neck pain, low back pain, cervical lordosis, scoliosis

## Abstract

We present a case report of a patient suffering from chronic low back pain (CLBP) and chronic non-specific neck pain (CNSNP), both of which were caused and complicated by a physically demanding occupation, a history of mixed martial arts, and lumbar scoliosis. Improvements in patient-reported outcomes (PROs) and radiographic findings were observed following conservative spine rehabilitation.

The patient, a 34-year-old male, had experienced chronic spine pain, particularly CLBP and CNSNP, for several years. He reported severe pain and increasing disability after a recent neck injury sustained while practicing jiu-jitsu. Radicular pain, along with numbness and tingling, was noted in the right upper extremity, extending to the first three digits, and there were also altered sensations and temperature changes in both feet. He described sharp, pinching mid-back pain and worsening disability due to the persistent pain, which led him to seek manual manipulative chiropractic spine therapy, though he reported little benefit from it. The patient had relied on over-the-counter pain medications for many years without achieving long-term pain and disability relief, and these medications were no longer used following treatment.

Chiropractic BioPhysics® (CBP®) spinal structural rehabilitation protocols were used to improve coronal and sagittal balance, as well as paraspinal muscular strength, addressing posture, mobility, and related aspects. These protocols include postural exercises, postural Mirror Image® traction, and postural spinal manipulative therapy. All PROs improved, with a near resolution of all initial symptoms of chronic spine pain. Outcomes measured included disability indices and health-related quality of life (HRQoL) indicators. Radiographic parameter improvements were significant, demonstrating improved coronal and sagittal balance as a result of the treatment. Following 30 in-office treatments, administered three times per week for 10 weeks, initial outcomes were reassessed. The patient then received 13 in-office treatments periodically over one year, and all initial outcomes were repeated. The improvements remained stable over time. A 26-month follow-up found that the improvements were sustained over a very long period without additional treatment after the 13-month examination. Chronic spine pain, specifically CLBP and CNSNP, is a significant source of suffering and contributes substantially to the global burden of disease. Improvement in HRQoLs, PROs, and objective spine parameters are desirable clinical outcomes.

Our case report documents objective improvement in lumbar scoliosis and spine pain, which is rare in conservative studies. This successful treatment of chronic pain with long-term follow-up contributes to the growing evidence supporting conservative, non-surgical treatments for CNSNP and CLBP. Successful management of chronic spine pain was observed in a patient undergoing CBP® treatment. The treatment was designed to address abnormal sagittal and coronal postural balance and radiographic abnormalities indicating spinal misalignment and reassess progress in PROs, as well as objective and subjective HRQoL measures, both following treatment and 13 months later. However, larger studies are needed to draw firm conclusions regarding the efficacy of this treatment for chronic pain.

## Introduction

We present the successful treatment of a patient suffering from chronic spine pain, specifically chronic non-specific neck pain (CNSNP), chronic low back pain (CLBP), and scoliosis, through a conservative spine rehabilitation program. The global burden of disease (GBD) is immense for musculoskeletal and spine pain disorders, with CLBP consistently reported as the leading cause of disabling pain worldwide. CNSNP is frequently cited as the fourth leading cause of disability globally, with low back pain being the primary cause [1-3]. Musculoskeletal spine pain significantly contributes to lost productivity, imposes a substantial financial burden on society, and leads to many years of living with disabilities. CNSNP and CLBP are major sources of suffering, making the availability of treatment options crucial not only for the affected patients but also for physicians, researchers, educational institutions, third-party payers, and governmental regulatory bodies.

There is a growing body of evidence supporting conservative therapies designed around measured biomechanical abnormalities in the spine [4]. Previous research has demonstrated that abnormal spine parameters increase the risk of dysfunction, pain, and disability [5-9]. The Chiropractic BioPhysics® (CBP®) spine rehabilitation technique includes protocols aimed at correcting abnormal spine parameters through specific Mirror Image® (MI®) exercises, which are designed to enhance paraspinal and postural muscle strength; MI® spine traction, which aims to deform the ligaments toward biomechanical normal models; and MI® spinal manipulative therapy (SMT) for addressing postural abnormalities [5-9]. These protocols have been studied across various conditions, including CNSNP, CLBP, and other spinal conditions [10,11]. The CBP® protocol was applied in this case report, with numerous outcome measures evaluated prior to treatment, reassessed following a set period of rehabilitation, and measured again 13 months later with home exercises and 15 in-office treatments administered periodically in the interim. A 26-month post-treatment long-term follow-up was conducted, with both objective and subjective outcome measures repeated.

## Case presentation

Patient history and clinical findings

A 34-year-old male had been suffering from chronic pain. The worst pain was found in the neck and low back, and the patient reported that the pain originated from a combination of very hard labor as a heavy equipment manufacturer in the mining industry. Additionally, he had a 12-year history of competitive rugby and a four-year history of martial arts. He had previously sought chiropractic care with no long-term benefit and only very short-term pain reduction. Four days prior to seeking treatment at a spine rehabilitation facility in Gillette, Wyoming, he sustained a neck flexion injury during jiu-jitsu. He reported neck pain with radiating pain from the neck to the right hand, coupled with tingling and minor loss of hand function. The pain was severe enough to affect his quality of sleep, and he was concerned it might impact his safety at work. He also reported pain on the left side of the ribcage with a pinching sensation in the mid-back.

His low back pain was concentrated near the sacroiliac joints bilaterally and throughout the lumbosacral region. The pain radiated from the low back to the right posterolateral region of the knee. The CLBP frequently caused cramping sensations in the calf, which were worse on the right side. The patient was also losing sensation in his feet bilaterally with temperature changes and frequently experienced cold feet. He reported frequent urination since contracting a bladder infection nearly a year prior to treatment. Additionally, he experienced epistaxis that interfered with his life, occurring twice per week.

Physical, orthopedic, and neurological examinations revealed hypoesthesia along the C6 dermatome on the right side and hypoesthesia along the L5 dermatome on the left. Deep tendon reflexes were slightly reduced when testing the patellar tendons bilaterally. Visual assessment of the cervical spine range of motion (ROM) indicated that all ROM was reduced and caused pain with all motions. Foraminal compression testing of the cervical spine was positive for neck pain and right arm radicular symptoms. Shoulder depression testing was positive for pain in the neck bilaterally, and cervical distraction resulted in a slight reduction in neck pain and fewer radicular symptoms on the right. Given the patient's history of high-level athletics and the physical nature of his occupation, his grip strength was greater than would be expected for his sex and age. Grip strength on the dominant right hand measured 145 lbs and 130 lbs on the left.

The visual coronal postural assessment found right head translation compared to the thorax (-TxH), coupled with left lateral flexion of the thorax (-RzT). There was also forward head posture or anterior translation of the head compared to the thorax (+TzH). The pelvis was rotated anteriorly on the right (+RyP). The leg-length radiographic analysis revealed a 5 mm discrepancy, with the left side shorter [12].

Radiographic findings

**Figure 1:**
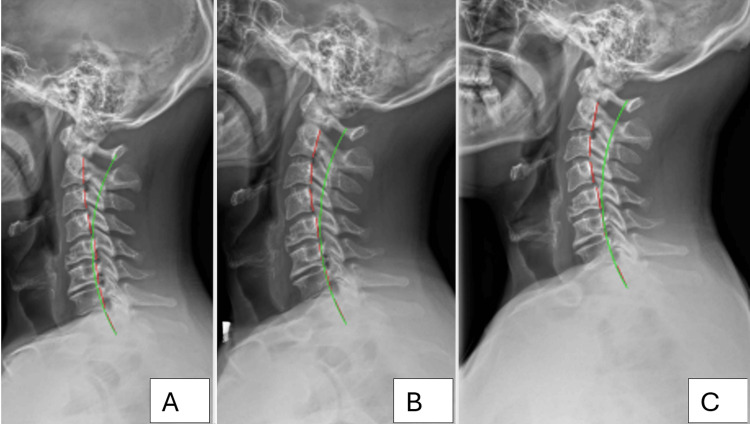
Lateral cervical radiographs. Panel A is the pre-treatment initial radiograph. Panel B is the post-treatment radiograph. Panel C is the 13-month long-term follow-up. The dotted red lines represent the HPTM analysis of the posterior body margin of the individual cervical spine segments. The green line is an ideal model of cervical lordosis for comparison of the patient’s parameters and a normal model. The spine parameters were digitized using PostureRay® software. HPTM: Harrison posterior tangent method

**Figure 2:**
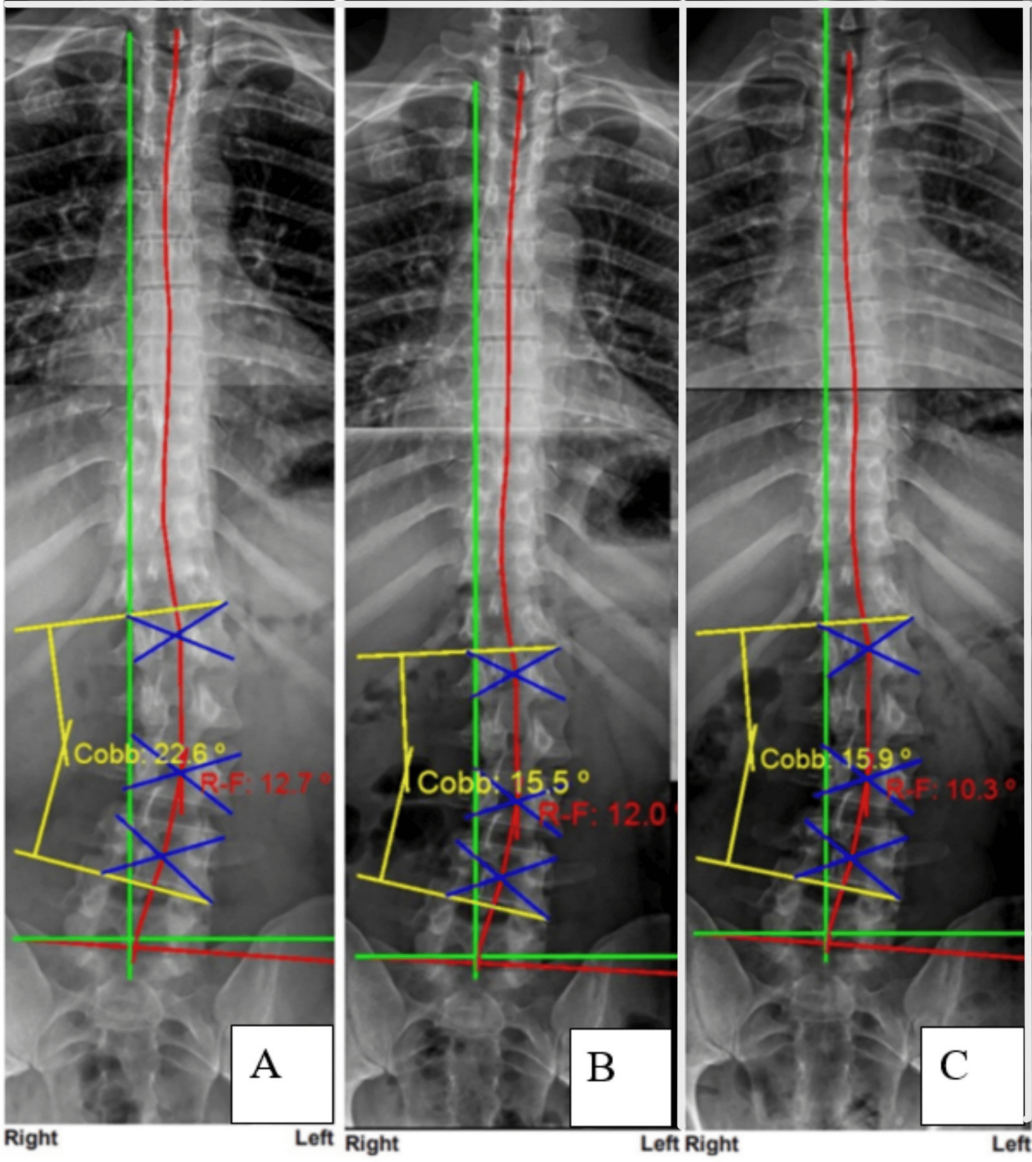
Anterior-posterior full spine radiographs. Panel A is the pre-treatment. Panel B is the post-treatment. Panel C is the 13-month long-term follow-up. The red line represents the Riser-Ferguson center-of-mass analysis of the coronal contour of the patient’s spine. The yellow lines represent the four-line Cobb analysis of scoliosis. The green line represents an ideal equilibrium model of the ideal spine. The results show improvements in the scoliotic curve and improvements in spine coronal balance and stability over the long term. All measurements were performed with PostureRay® software.

Upright spine radiographs were obtained by a licensed physician and were taken in accordance with all federal and state guidelines. Anterior-posterior and lateral radiographs were taken of the cervical, thoracic, and lumbopelvic spines. The radiographs found no pathologies requiring referral for additional testing. The radiographs were evaluated for biomechanical parameters using the computerized digitization tool PostureRay® (PostureCo®, Inc., Trinity, Florida, USA) [13]. The results revealed multiple abnormal spine parameters. Disc and bone degenerative changes were visualized at the C4/C5 and C6-C7 levels, and the C5 segment appears deformed from a possible prior flexion injury. Lateral cervical radiography found the overall cervical lordosis absolute rotation angle (ARA C2-C7) to be -11.4°, compared to the normal range of -20° to -40° using the Harrison posterior tangent method (HPTM) (Figure 1) [8]. The anterior translation of the head measured 17.6 mm compared to a normal measurement of 0 mm. Coronal films of the lumbopelvic spine showed a moderate to large thoracic translation to the left compared to the pelvis, measuring 16.9 mm versus a normal measurement of 0 mm. This translation was coupled with a levoscoliosis measuring 22.6° using a four-line Cobb L1-L4 analysis (Figure 2). The sagittal lumbar spine film showed an increased lumbar lordosis ARA from L1-L5, measuring -54.5°, compared to an ideal value of -40° [14].

Initial pain and disability assessments

The Quadruple Visual Analog Score (QVAS) was used, where 0/100 indicates no pain and 100/100 indicates the worst pain imaginable (Table 1). The initial score for cervical pain at the time of evaluation was 40/100, average pain was 60/100, pain at its best was 20/100, and pain at its worst was 80/100. The composite total score at the initial exam was 60/100. The initial QVAS for thoracic pain at the time of the exam was 50/100, average pain was 60/100, pain at its best was 40/100, and pain at its worst was 70/100, resulting in a composite score of 60/100. The initial QVAS for the lumbar spine was 30/100 for current pain, average pain was 40/100, pain at its best was 10/100, and pain at its worst was reported as 80/100, with a composite score of 50/100.

**Table 1:** QVAS results. The patient's reported pain scores were assessed at the initial examination, post-treatment re-assessment, and 13-month long-term follow-up. All scores improved following treatment and were stable long-term. The cervical, thoracic, and lumbar scores were assessed at each examination. QVAS: Quadruple Visual Analog Score

Evaluation	QVAS cervical				
Date	Current pain	Average pain	Pain at best	Pain at worst	Total
2/28/2022	4\10	6\10	2\10	8\10	60\50
5/11/2	0\10	1\10	0\10	3\10	13\50
6/13/2023	1\10	1\10	0\10	2\10	13\50
4/2/2024	0\10	0\10	0\10	2\10	2\50
Overall change	4\10	6\10	2\10	6\10	58\50
Evaluation	QVAS thoracic				
Date	Current pain	Average pain	Pain at best	Pain at worst	Total
2/28/2022	5\10	6\10	4\10	7\10	60\50
5/11/2023	1\10	1\10	1\10	2\10	13\50
6/13/2023	1\10	1\10	0\10	2\10	13\50
8/2/2024	0\10	1\10	0\10	1\10	10\50
Overall change	5\10	5\10	4\10	6\10	50\50
Evaluation	QVAS lumbar				
Date	Current pain	Average pain	Pain at best	Pain at worst	Total
2/28/2022	3\10	4\10	1\10	8\10	50\50
5/11/2023	1\10	2\10	1\10	4\10	23\50
6/13/2023	2\10	2\10	1\10	4\10	27\50
8/13/2023	1\10	1\10	1\10	1\10	10\50
Overall change	1\10	3\10	1\10	7\10	40\50

The Neck Disability Index (NDI) outcome measure was calculated at 28/100, indicating severe disability. The Revised Oswestry Disability Index (RODI) at the initial evaluation measured 26/100, indicating severe disability due to low back pain. A short-form 36-question health status questionnaire (SF-36) was used to assess overall health and well-being (Table 2). The scoring ranges from 0 to 100, with 0 representing no function and 100 representing perfect function with no interference. The initial health perception measurement was 55/100, physical functioning was 85/100, physical impact due to pain was 0/100, emotional impact of pain on function was 67/100, social function was 50/100, mental health function was 52/100, overall bodily pain was 23/100, and energy/fatigue impact was 35/100.

**Table 2:** Results of the SF-36 demonstrating improvement across multiple health categories from the initial assessment, changes at re-examination, and the one- and two-year follow-up changes. The results indicate the treatment improved these health parameters and persisted in long-term assessment. SF-36: short-form 36-question health status questionnaire

Date	Health perception	Physical function	Physical ability	Emotional health	Social function	Mental health	Bodily pain	Energy/fatigue
2/28/2022	55	85	0	67	50	52	23	35
5/11/2022	77	100	100	100	100	88	80	65
6/13/2023	82	100	100	100	100	80	68	30
8/2/2024	72	100	100	100	75	68	68	50
Overall change	17	15	100	33	25	16	45	15

Treatment protocols and frequency

**Figure 3:**
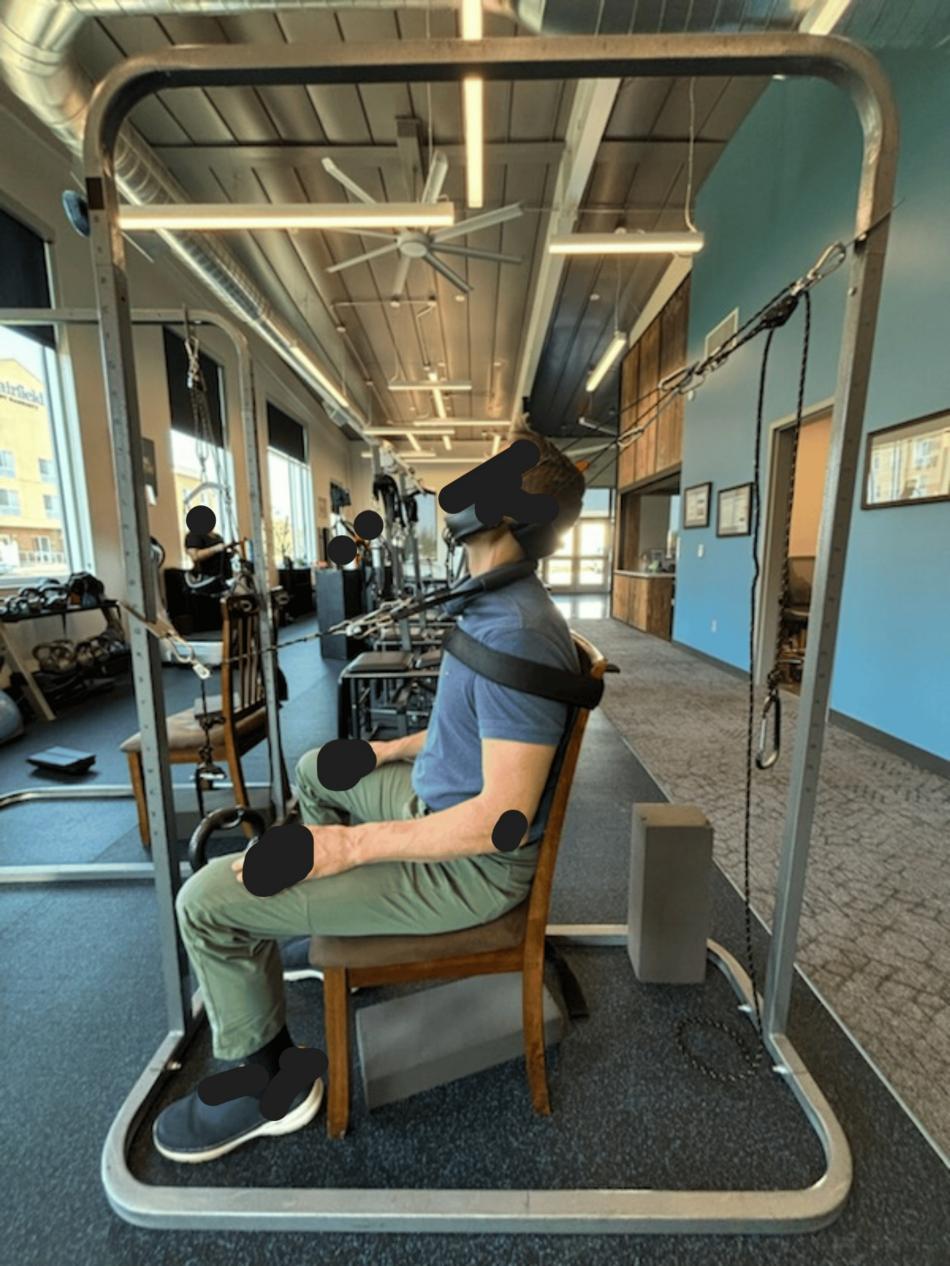
MI® cervical spine two-directional traction. The patient is seated, and the thorax is gently restrained with a Velcro strap to prevent movement. Two loads are applied to the patient: (1) a superior-posterior load to increase some spine unloading in an extended and posteriorly translated position and (2) a posterior-to-anterior load in a slight caudal orientation. The traction aims to cause sustained loading while the cervical lordosis is increased. The duration and intensity increase throughout the treatment program. MI®: Mirror Image®

**Figure 4:**
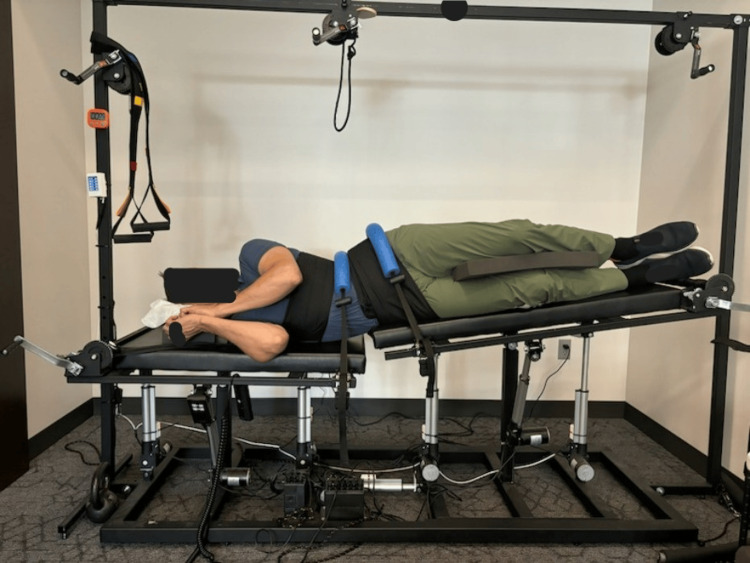
Robo-TracTM coronal MI® traction designed to address the abnormal left coronal balance seen on the anterior-posterior radiograph with a significant right lateral translation of the torso compared to the pelvis and lower extremity. A firm strap is placed at the apex of the scoliosis, and the table is able to slightly distract the lumbar spine to unload the joints and increase the focus in the MI® position of the abnormalities found with the PostureRay® digitization software. MI®: Mirror Image®

The subject received CBP® structural spinal rehabilitation protocols in-office for 30 treatments over 10 weeks, with a frequency of three times per week. The patient was treated with MI® SMT, MI® postural exercises, and MI® traction. The PostureRay® digital assessment of the radiographs was used to determine the appropriate MI® protocols, including the vector of force for the traction. Initial MI® traction began with a three-minute duration, increasing to 15 minutes per session. This traction involved the patient seated in a chair that encouraged an upright thoracic posture, with the center of the thorax positioned over the pelvis. The patient experienced a cephalad and posterior traction force at a 20° vector, starting with 10 lbs and progressing to 12 lbs. Additionally, there was a posterior-anterior load at a 10° angle, beginning at 10 lbs and increasing to 20 lbs. The purpose of this traction was to gradually stretch the viscoelastic ligamentous structures in a lordotic direction, while moving the upper cervical spine into an extended position, exceeding the threshold of hysteresis to induce a permanent change in sagittal balance and lordosis, and to unload the central and peripheral nervous system (Figure 3). A second traction, targeting abnormal coronal plane spine parameters, was applied with the patient in a right recumbent position on a specialized traction table. This setup allowed the MI® position of right lateral thoracic translation (-TxT) coupled with a left-to-right lateral force vector at the apex of the thoracolumbar curve. A foam block was used to keep the knees separated and the legs parallel. This traction began at three minutes, progressing to 15 minutes or to tolerance, and utilized the Robo-Trac™ traction device (Advanced Spinal Rehab, Middletown, NY, USA) (Figure 4).

**Figure 5:**
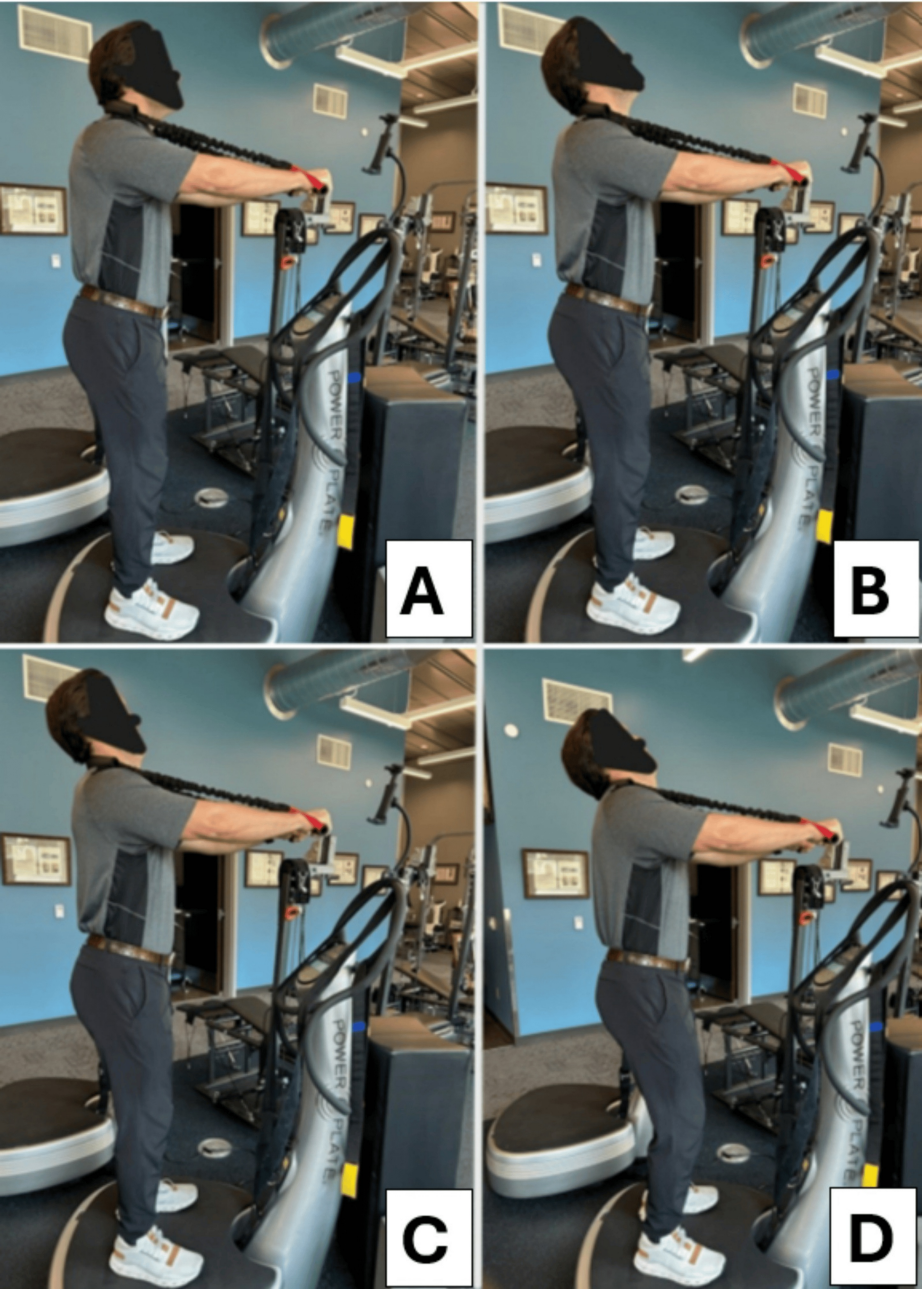
The FLIPTM protocol to increase lordosis. Panel A shows the patient anteriorly translating the head forward to increase lower cervical flexion. Panel B shows the patient extending the head against the ProLordoticTM resistance. Panel C shows the patient posteriorly translating the head. D shows the patient posteriorly and caudally translating the head to increase lordosis. The patient is performing the exercise under the influence of whole-body vibration on the Power Plate®. FLIPTM: Fedorchuk lordosis-inducing protocol

Additionally, the patient performed spine muscle strengthening exercises using the ProLordotic™ cervical spine exerciser (Circular Traction, LLC, Huntington Beach, CA, USA) under whole-body vibration on the Power Plate® (Power Plate Performance Health Systems, LLC, Northbrook, IL, USA). The patient followed the Fedorchuk lordosis-inducing protocol (FLIP™), which consisted of (1) anterior head translation to increase flexion of the lower cervical segments, (2) extension to enhance cervico-cranial angulation of the upper cervical spine, (3) posterior head translation to improve sagittal balance, and (4) posterior-caudal/inferior translation of the head to increase cervical lordosis (Figure 5). This four-step exercise has previously been shown to increase lordosis and should be confirmed via radiography at the end position to determine if the combination is appropriate for the individual, that is, if the cervical lordosis is improved [10]. The patient performed the exercises for 5-10 seconds, starting with 30 repetitions, and was encouraged to increase to 50 repetitions daily with a sustained hold of no more than 15 seconds. The patient reported no increase in symptoms as a result of any of the exercises, MI® SMT, or sagittal and coronal traction.

**Figure 6:**
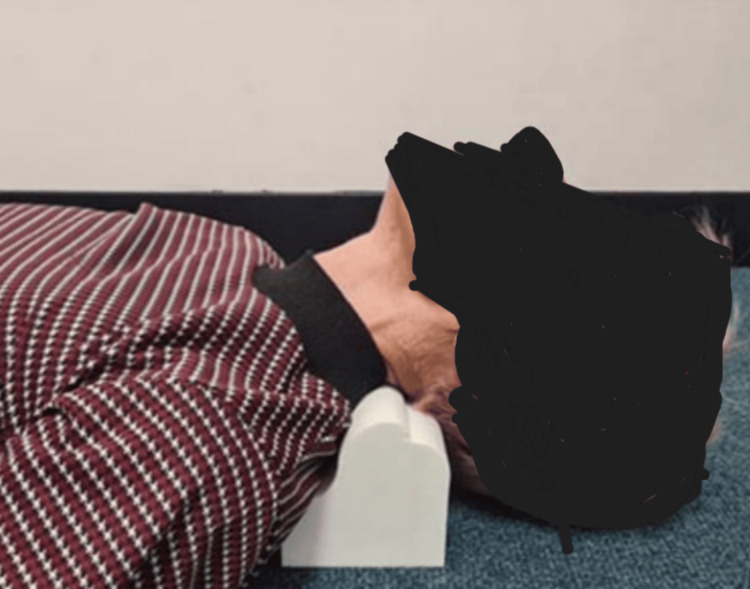
DCTO. A model representation of the Denneroll® device being applied to the region that matches the placement in this case; low placement due to the reduction in the lordosis in the lower cervical spine worsening superiorly. The traction was utilized approximately two times per week by the patient for the 13 months following the treatment re-assessment and long-term follow-up. The duration is initially mild with two to three minutes on the orthotic and increases with tolerance to 15-20 minutes. DCTO: Denneroll® cervical traction orthotic

The patient received in-office treatment and was also provided with home treatment protocols, including the four-step FLIP™ cervical spine postural exercises for improving sagittal balance, ergonomic postural education, and a home prescription for a lordosis-inducing device to assist with in-office traction (Denneroll® cervical traction orthotic (DCTO)) (Figure 6). The patient was instructed to perform the postural exercises three to five times per week, with each repetition lasting 15 seconds and as many tolerable repetitions as possible. The DCTO treatment was initiated at two to three minutes and gradually progressed to 15-20 minutes, up to five times per week. Patient compliance was high for both in-office therapy and home care. The initial evaluating physician, who performed the assessments, conducted the re-evaluation, including spinal radiography, all outcome measures, and QVAS re-assessment. No adverse events were reported by the subject. The patient reported high satisfaction with the in-office treatment and its outcomes and provided both verbal and written consent to publish the treatment case, including all pictures.

Results

Post-treatment Subjective and Objective Patient-Reported Outcome Measure Findings

The patient underwent 30 in-office treatments, which included MI® exercises, MI® traction, and MI® postural SMT. Following the treatment program, all initial subjective and objective outcome measures were reassessed by the same physician who conducted the initial evaluation. A comparison of these measures showed improvements in symptoms and reduced dysfunction compared to baseline data. The patient reported significant improvements in CNSNP, CLBP, and related disabilities, as well as in posture and the ability to perform activities of daily living (ADLs). Specifically, the patient noted a 90% improvement in CNSNP, a 90% improvement in radicular and right arm abnormal sensation, and complete resolution of pain. The left thoracic ribcage pain improved by 80%, while lumbosacral pain and thoracolumbar CLBP were reported to have improved by 85%. Radicular right leg and posterolateral knee pain improved by 90%, and all leg cramping was resolved. The abnormal sensation bilaterally in the feet, including temperature intolerances and cold feelings, was resolved. Frequent epistaxis also resolved, and consistent bladder infections, compared to the prior year, were reported to have improved by 80%.

The physical examination revealed significantly fewer positive orthopedic tests compared to the initial evaluation. Grip strength improved and equalized, and all dermatomal testing was now negative. Prior cervical compression testing was negative for any axial or peripheral pain or pain referral syndromes. The only positive patient-reported abnormality was continued hypoesthesia at the L5 dermatome with pinwheel testing. All reflex testing was normal. All visual ranges of motion for the cervical, thoracic, and lumbar spine, assessed for pain, were within normal limits (WNL) and pain-free. Subjective improvements were consistent with objective outcome measures.

The cervical QVAS at re-evaluation following initial treatment showed dramatic improvement, with current pain at the time of the exam reduced to 0/100, average daily pain reduced to 10/100, pain at its best reduced to 0/100, and pain at its worst measuring 30/100, indicating very little disability due to neck pain following treatment. The composite QVAS score was 13/100. Thoracic QVAS measurement for pain in the thoracic spine also showed improvement, with current pain measuring 10/100 compared to 50/100 initially, average pain 10/100 versus 60/100, pain at its best 10/100 compared to 40/100, and pain at its worst 2/10 versus severe 70/100, resulting in a composite score of 13/100 compared to a severe 60/100 initial score. Lumbar spine QVAS similarly demonstrated improvement across all outcome measures, with pain at the time of evaluation measuring 10/100, average pain 20/100, pain at its best 10/100, and pain at its worst 40/100. The composite score improved from an initial 50/100 to 23/100, indicating greater than 50% improvement (Table 1).

**Table 3:** NDI and RODI results of the initial, post-treatment, and 13-month long-term follow-up. The initial findings found moderate to severe disability with a significant reduction to mild disability at the post-treatment follow-up and only a slight return toward baseline at long-term follow-up. NDI: Neck Disability Index, RODI: Revised Oswestry Disability Index

Disability indices	NDI	RODI
Date		
2/28/2022	28/50	26/50
5/11/2022	6/50	10/50
6/13/2023	10/50	14/50
8/2/2024	8/50	16/50
Overall change	20/50	10/50

NDI demonstrated an improvement from an initial score of 28/50 to 6/50 at the post-treatment assessment. Further improvements were objectively measured with the RODI, which showed an initial score of 26/50, reducing to 10/50 at the post-treatment assessment (Table 3). The SF-36 revealed significant improvements across health perception, physical impact of pain, emotional function, bodily pain, and energy level/fatigue (Table 2).

Post-treatment Radiographic Outcome Findings

Following 30 in-office treatments, the patient was not treated for more than 24 hours and was instructed to avoid performing any home exercises prior to the re-assessment. This pause in care ensures that the results of the radiographs are not affected by any residual effects of the previous treatment. The post-treatment radiographs were acquired by the same physician who initially captured the images, with the same patient instructions to ensure neutral positioning without any external interference or examiner bias in patient positioning.

All films were digitally measured using the machine-learning-assisted radiographic parameter mensuration software PostureRay®. Improvements were observed in the coronal and scoliotic curves, and pre- and post-treatment images reveal enhancements in segmental angulation. The most notable and significant change was observed in the cervical lordosis, with an initial angle of -11.4° improving to -30.6°. Additionally, there was an improvement in the overall lordosis at L1-L5, with an angular change of 12.2°. Lumbar coronal angular improvements were most pronounced in the lumbar spine, with the four-line Cobb measurement improving from an initial 22.6° to 15.5° at re-assessment. Thoracic translation improved by 6.4 mm (Figures 1-2).

One-Year Follow-Up Findings

A 13-month follow-up was performed during which the patient reported frequent (three to five times per week) engagement in home exercises and use of the DCTO, in addition to receiving 15 periodic in-office treatments comprising exercise, traction, and MI® SMT. At the follow-up assessment, all initial examinations and both subjective and objective outcomes were repeated by the same physician who conducted the initial and post-treatment examinations. The patient continued to experience minimal spine pain symptoms and no symptoms that prompted initial care and reported high satisfaction with both in-office and home care.

Patient-reported outcomes (PROs) and subjective findings indicated continued improvement more than a year after the cessation of frequent treatment. Neck pain remained 90% improved from baseline; there was no radiculopathy affecting function, and the right arm pain and sensory abnormalities present at the initial exam remained 90% improved. The patient reported that pain no longer interfered with sleep and was 70% improved from the initial evaluation. Left ribcage and thoracic pinching pain remained 70% improved. Low back pain and pain concentrated in the sacroiliac joints were reported as 70% improved compared to the initial assessment. Low back pain leading to calf cramping continued to improve, with a reported improvement of 70%. Only very mild temperature abnormalities were reported, with 80% of the cold sensation improving bilaterally since the initial evaluation. The NDI showed minimal neck disability compared to the initial evaluation, measuring 10/50 compared to 26/50 initially. The RODI for low back pain disability slightly returned to baseline, measuring 14/50 compared to 26/50 initially (Table 3).

Cervical, thoracic, and lumbar QVAS demonstrated continued improvement, with the lumbar composite remaining stable and the cervical and thoracic QVAS improving slightly (Table 1). Epistaxis continued to be resolved, and the patient reported only a very mild bladder infection once in the past year, attributed to reduced inflammation and improved nervous system function. Regarding the epistaxis and bladder issues, the patient did not report any other lifestyle changes besides a much greater emphasis on posture, spine, postural muscles, and ergonomics in daily life. The SF-36 indicated continued or maintained improvement in categories such as health perception, physical functioning, physical ability due to pain, emotional health, and social functioning; only mental health, bodily pain, and energy/fatigue showed very slight reductions compared to reassessment, though they still improved from baseline (Table 2). Overall, both subjective and objective outcomes demonstrated continued improvement from baseline, with a significantly reduced treatment frequency over the 13 months.

Two-Year Follow-Up Findings

The patient returned for a follow-up 26 months and three weeks after the final post-treatment assessment. There had been no in-office treatment since the first 13-month follow-up, and only sporadic DCTO and ProLordotic™ FLIP™ exercises were reported. He reported that overall improvement was well-maintained; CNSNP was 100% improved, and initial upper extremity pain was 90% improved, including the prior numbness and tingling radiculopathy. Initial mid-back pinching pain was 80% resolved, and lower extremity cramping and cold feet were 90% improved. Epistaxis was 80% resolved, and bladder-related frequent urination was 70% improved. The sagittal and coronal postural evaluations were maintained since the post-treatment examination. The trapezius muscles were slightly tender with deep palpation bilaterally. No positive orthopedic or neurological tests were found. Bilateral hand grip strength testing was measured at 140 lbs, which is above normal for age and sex. All cervical and lumbar ROM assessments for pain were WNL. Only right head rotation caused slight cervico-thoracic discomfort. NDI calculated a well-maintained improvement, measuring 8/50; RODI also demonstrated maintained improvement, measuring 10/50. Total cervical QVAS improvement was 58/100, thoracic QVAS improvement was 50/100, and lumbar QVAS improvement was 40/100. All results demonstrate long-term improvement and stability of reported pain (Table 1).

**Figure 7:**
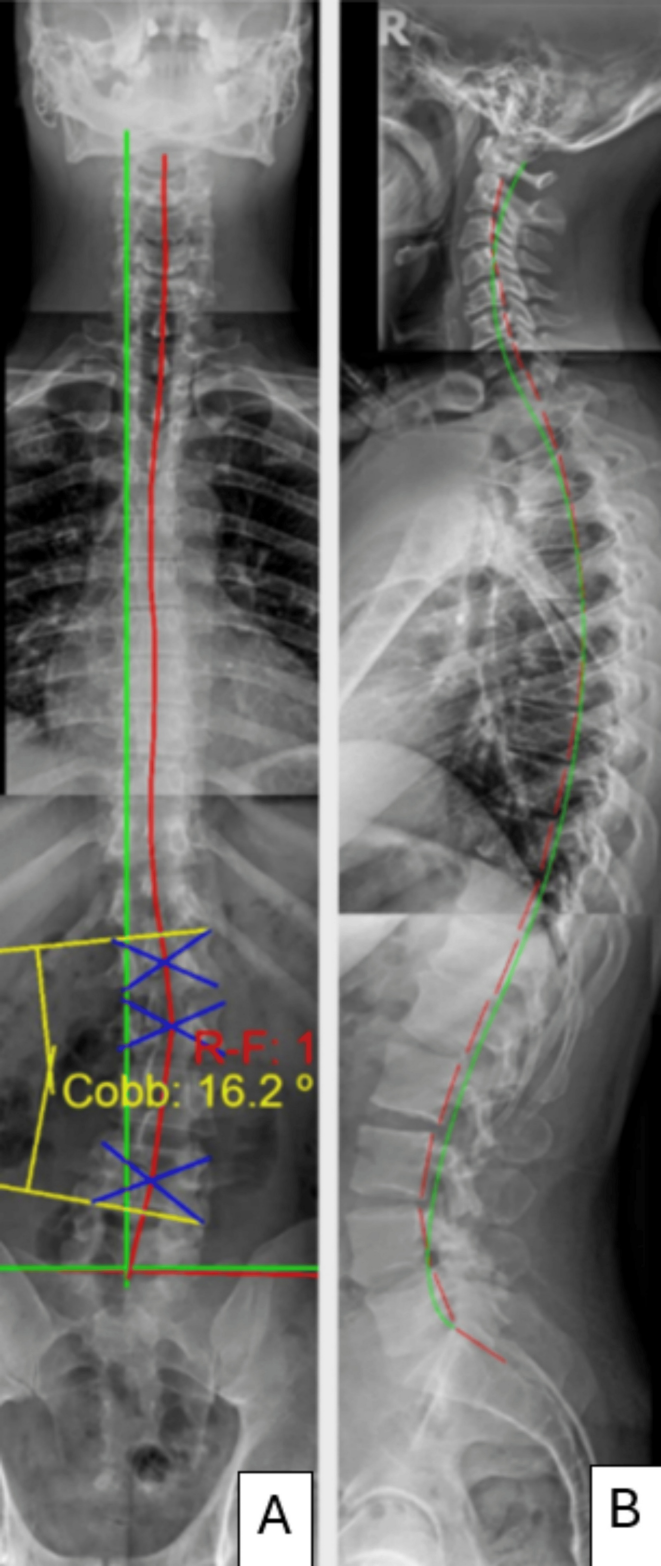
Panel A shows the patient’s two-year long-term anterior-posterior full spine radiograph, showing the patient's coronal balance is well preserved in the long term. Panel B shows the patient’s full spine lateral radiograph also shows well-maintained sagittal balance, cervical and lumbar lordosis, and thoracic kyphosis. The green line is the ideal spine model, and the red dashed line traces the patient's posterior vertebral body tangents.

Radiographic findings revealed well-maintained sagittal balance, stable lumbar scoliosis, and coronal balance. Most notably, the cervical lordosis was deep, measuring -36° from C2-C7 using the HPTM. Thoracic kyphosis measured 41.6° from T1-T12, which is only 5.5% off from the ideal angulation of 44°. Lumbar lordosis measured -44° from L1-L5, showing nearly normal angulation (ideal = 40°). The four-line Cobb measurement of lumbar scoliosis was 16.2°, demonstrating maintained improvement from the post-treatment evaluation (Figure 7).

## Discussion

This case documents the reduction of lumbar scoliosis and the increase in cervical lordosis, as well as improvements in other radiographically measured biomechanical parameters that corresponded with enhancements in pain intensity, disability, and quality of life measures in a 34-year-old male following a conservative rehabilitation program. All outcome measures were repeated at each examination. The initial results were also maintained at the 13- and 26-month follow-up examinations.

Spine parameter abnormalities and altered alignment appeared to be a significant source of the patient's lack of improvement with previous pharmacological, over-the-counter, and traditional chiropractic treatments. Altered spine configuration has been discussed as a potential correlation or causative factor in CNSNP, and abnormal spine parameters are associated with numerous studies of CLBP and chronic spine pain, often leading to adult spine deformity [1,3,4,15-18]. CNSNP and CLBP warrant a triage of treatment options that should include conservative therapeutics and progress to more invasive options if conservative treatments fail to improve both objective and subjective outcome measures. Musculoskeletal conditions, including CNSNP and CLBP, represent a significant cause of disability and contribute substantially to the GBD [1-4]. Patients suffering from these conditions are frequently unable to perform at the level of their peers in terms of work and ADLs.

Although several studies over many decades have reported the biomechanical causes of CNSNP and CLBP, recent research has highlighted the contribution of biopsychosocial factors that should not be dismissed. However, it is detrimental to the patient if the diagnosing physician ignores the significant biological and biomechanical contributions and relies solely on psychosocial factors. For instance, a recent commentary by Harrison et al. reported: “Proponents of the stronger role that the 'psycho-social' part of the equation plays in spine conditions often fail to acknowledge that recent systematic literature reviews with meta-analysis have identified a clear controversy regarding the quality and true impact that fear-avoidance, pain-catastrophizing, and 'psycho-social' model elements play in individuals with chronic musculoskeletal pain disorders” [18]. The literature clearly indicates that it is inappropriate to make any diagnosis or intervention recommendations for abnormal spine conditions without a biomechanical analysis, including radiographs.

Chronic spine pain and associated conditions require proper diagnosis by clinicians and first-contact providers. Emergency rooms and primary care providers are the most likely to encounter spine pain, so correct triage and referral should include a comprehensive evaluation with multiple subjective and objective outcome measures. Outcome tools such as the QVAS, NDI, RODI, SF-36, and other measures specific to the condition should be considered standard protocol to determine the necessity of care, therapeutic intervention options, and the results of the intervention [19-21]. Patients undergoing pharmacological, conservative, therapeutic, or invasive surgical treatments should have appropriate outcome measures completed before any recommendations for care and future treatment. Prior trauma and a history of spine pain and associated conditions significantly predispose individuals to a reduction in quality of life due to pain and dysfunctional ADLs. Furthermore, these individuals are at much greater risk of future disability due to these conditions. Proper documentation and outcome measures not only assist patients in their treatment but also aid physicians, third-party payors, and regulatory boards in confirming the necessity of care and comparing outcomes when different treatments are applied [1-3].

Complications for CNSNP, such as female gender, advanced age, and prior trauma, must be reported. It is requisite that CLBP is appropriately evaluated because CLBP is the greatest contributor to GBD, and early, effective, repeatable, and economical interventions help reduce both suffering and financial burden [1]. Prior studies have demonstrated that buckling in the coronal and sagittal planes of the spine leads to accelerated fibrosis of repair and increased infiltration of nociceptive afferentation from nerve-ending sprouting [22]. Spinal trauma, initially and further if the pain becomes chronic, produces inflammatory exudates and abnormally loads the tissues, accelerating degeneration and tissue deformation. Interventions, whether conservative or surgical, improve spine parameters and coronal and sagittal balance. Proper diagnostic procedures are essential to ensure accurate diagnosis and appropriate treatment options [22-23].

Radiography has been firmly established as the gold standard for accurately diagnosing spine conditions. Recent advances in X-ray acquisition have improved image quality and raised safety concerns. Furthermore, research on the potential harms of X-ray radiation from diagnostic imaging has revealed misconceptions that must be addressed. For instance, an extensive review by Bondy found: “Overall evidence suggests that very low dose rates have often been found to increase lifespan through a mechanism likely to involve adaptive responses. An inverse dose rate response has been found in studies performed below levels of natural background radiation” [24]. Spine radiography is safe, and methods for assessing measured parameters have dramatically improved with the use of machine learning and artificial intelligence [13]. Commentaries and limited or brief reviews claiming that spine radiography is unnecessary or poses an increased risk are unfounded. Regulatory boards and guidelines should not consider these selective reviews and commentaries in their decisions regarding the judicious and important use of X-rays for spine diagnosis [25]. A very recent systematic review of the literature has further confirmed the reliability and validity of spine radiography measurements [26].

The treatment protocol employed CBP® methods to decrease a lumbar scoliosis curve in an adult. Adult scoliosis, referred to as "de novo," has a relatively high prevalence [27]. Importantly, it is uncommon to find literature documenting a reduction in adult scoliosis [28]. One case series reported four adults experiencing a reduction in scoliosis curves ranging from 7° to 14° after CBP® treatment protocols. This case demonstrated a 7.1° decrease, which was maintained for two years. This degree of change is considered significant, as it exceeds the 6° minimum cut-off required to claim a true improvement in scoliosis, as first suggested by Carman et al. [29]. The success of this protocol has been documented in previous case reports/series as well as randomized controlled trials. The results from this case are consistent with those of prior studies and contribute to the growing body of evidence supporting this conservative approach [5-9,11].

Limitations of this case include that it involves only a single patient; thus, generalized conclusions regarding these results cannot be made. Furthermore, although there was a two-year follow-up, the long-term maintenance of spine and symptom improvements remains unknown. It is logical to assume that ongoing maintenance treatments may be necessary.

## Conclusions

The reduction of lumbar scoliosis in an adult is possible, and CBP® treatment can be directed at multiple spinal areas simultaneously to improve both sagittal and coronal plane spinal deformities. Furthermore, the improvement in spine and posture was consistent with other reports in the literature and corresponded with enhancements in pain intensity, disability, and quality of life measures, which were maintained at long-term follow-up. This case provides clinicians and researchers with additional evidence for the potential effectiveness of conservative treatments for spinal conditions.
